# Revealing brain mechanisms of mTOR-mediated translational regulation: Implications for chronic pain

**DOI:** 10.1016/j.ynpai.2018.03.002

**Published:** 2018-03-21

**Authors:** Chulmin Cho, Vassilia Michailidis, Loren J. Martin

**Affiliations:** a Department of Psychology, University of Toronto Mississauga, Mississauga, ON L5L 1C6, Canada; b Deptartment of Cell and Systems Biology, University of Toronto Mississauga, Mississauga, ON L5L 1C6, Canada

**Keywords:** mTOR, Pain, Multiple sclerosis, Depression, Alzheimer’s disease, Parkinson’s disease

## Abstract

In the spinal cord, altered protein transcription and translation have received a lot of recent attention for their role in neural plasticity, a major mechanism leading to the development of chronic pain. However, changes in brain plasticity are also associated with the maintenance of pain symptoms, but these cellular mechanisms remain less clear. The mechanistic/mammalian target of rapamycin (mTOR) is a master regulator of protein synthesis, and controls several neuronal functions, including neural plasticity. While aberrant changes in mTOR signaling are associated with sensitization of the pain pathway (sensory neurons and spinal cord), there are various nervous system diseases that have pain as a comorbidity and altered mTOR activity in the brain. Here, we provide a brief review of mTOR changes in the brain that are associated with some neurological disorders and focus on how these changes may be relevant to the pain of the underlying condition and chronic pain itself.

## Highlights

• mTOR is a major regulator of protein translation.
• mTOR serves an important role in neural plasticity.
• mTOR signalling in the brain as a pathology for neurological disorder is known.
• mTOR signalling in the brain as a chronic pain mechanism is understudied.

## Introduction

Pain is a complex sensory and emotional experience that motivates the withdrawal or escape from damaging situations. Pain as a motivator is useful in that it allows for the protection of a damaged body part, so that proper healing may occur. Most pain resolves once the damaging or noxious stimulus is removed, but in some instances, pain persists despite the apparent healing of an initial inciting injury. This type of pain, referred to as chronic pain, imposes a profound healthcare burden that not only affects the individual, but also permeates throughout all avenues of social, work, and family life. The etiology behind pain chronification remains elusive, but it is accompanied by changes in pain sensitivities and thresholds that are expressions of neural plasticity – an activity-dependent alteration in structure and function of neurons (Woolf and Salter, 2000). These long-lasting changes in neural plasticity are dependent on protein translation driven by the mRNA that resides inside cell bodies, axons and dendrites (Fernandez-Moya et al., 2014, Linden, 1996). In support of this, inhibition of protein synthesis in the spinal cord prevents central sensitization by disrupting the late phase of spinal long-term potentiation (LTP), a form of neural plasticity, where synaptic potentials are enhanced (Hu et al., 2003). Since many diseases of the brain are associated with aberrant plasticity, often caused by dysfunctional protein translation, we suspect that when these changes occur in pain-associated areas that this may lead to pain symptoms. This suggests that elucidating the mechanisms behind aberrant protein translation in the brain may lead to novel insights into delineating pain processing and pain chronification.

The mechanistic/mammalian target of rapamycin (mTOR), a major regulator of protein translation serves an important role in neural plasticity (Banko et al., 2005, Costa-Mattioli et al., 2009) with accumulating data indicating that it is involved in nociceptive processing (Geranton et al., 2009), and possibly chronic pain (Melemedjian et al., 2011). mTOR is a serine/threonine protein kinase that belongs to the family of phosphatidylinositol 3-kinase (PI3K)-related kinases. It is highly conserved across eukaryotes (Di Domenico et al., 2018), and forms two distinct complexes: the rapamycin-sensitive mTOR complex 1 (mTORC1) and rapamycin-insensitive mTORC2 (Hay and Sonenberg, 2004, Laplante and Sabatini, 2012). Regulation of protein translation is predominantly carried out by the mTORC1 complex (Wang and Proud, 2006), consisting of raptor (regulatory associated protein of TOR), mLST8 (mammalian Lethal with Sec13 protein 8) and PRAS40 (proline-rich Akt substrate of 40 kDa) amongst other subunits (Aylett et al., 2016). mTORC2 is comprised of mLST8, rictor (rapamycin-insensitive companion of mTOR), mSin1 (mammalian stress-activated protein kinase-interaction protein 1) and other subunits (Switon et al., 2017). In particular, mTORC1 is known to regulate four proteins that participate in either initiation of protein translation or the rate of translation via phosphorylation: eukaryotic initiation factor 4E-binding protein 1/2 (4E-BP1/2), eukaryotic initiation factor 4B (eIF4B), p70- ribosomal S6 protein kinase 1/2 (S6K1/2) and ribosomal S6 proteins (S6) (Lutz et al., 2015).

The majority of studies that have examined the role of mTOR in pain have been focused at the level of the spinal cord where mTOR expression is found in superficial dorsal horn and in dorsal root ganglia (DRG) neurons (Xu et al., 2010). In the dorsal horn and DRG, increased levels of phosphorylated mTOR were found in response to cancer-related, inflammatory and neuropathic pain including spinal cord injury (Liang et al., 2013, Lucas and Lipman, 2002, Lutz et al., 2015, Melemedjian et al., 2011, Shih et al., 2012, Wang et al., 2016, Xu et al., 2011, Zhang et al., 2013). Further, inhibition of mTOR using rapamycin reduced neuronal hyperexcitability in the dorsal horn and antinociception in animal models of inflammatory and neuropathic pain (Asante et al., 2009, Asante et al., 2010, Banko et al., 2005, Costa-Mattioli et al., 2009, Geranton et al., 2009, Liang et al., 2013, Obara et al., 2011, Price et al., 2007, Shih et al., 2012, Xu et al., 2011).

The pathological changes related to pain, especially persistent pain, are not limited to the level of spinal cord and there is evidence that alterations in brain plasticity are associated with pain sensitivity (Eto et al., 2018, Hashmi et al., 2013, Lee et al., 2018, Malfliet et al., 2017). These changes, in turn, lead to maladaptive downstream processing from the brain that may result in chronic pain (Bushnell et al., 2013). A major contributor to these pathological brain changes is known to occur at the level of protein translation, making the investigation of translational control in brain mechanisms of chronic pain of critical value. In line with this, mTOR is ubiquitously expressed in the brain where it regulates structural and functional components of neurons and glial cells (Bockaert and Marin, 2015). Importantly, altered mTOR activity within sensory or affective brain regions would have a great impact on pain processing and symptoms. However, the role of mTOR in the brain with respect to chronic pain remains understudied. Since dysfunctional mTOR signaling is found in a wide array of brain disorders that have pain as a comorbidity, we examine various neurological disorders where mTOR changes in the brain are well-documented and highlight the implications of these changes for understanding pain.

## Multiple sclerosis

Multiple sclerosis (MS), is an autoimmune disorder characterized by scattered areas of lesions or plaques in the central nervous system (CNS); demyelination, microglia activation, gliosis, and varying degrees of axonal damage leading to chronic neuroinflammation (Russo et al., 2016). A number of studies have shown that glial cells (microglia and astrocytes) become activated in rodent models of MS, or experimental autoimmune encephalomyelitis (EAE) (see Mallucci et al., 2015 for a review) and recently, a direct role of mTOR in the modulation of glial functions has been recognized (Dello Russo et al., 2013). Moreover, a direct link between mTOR phosphorylation and microglial pro-inflammatory activation has been found in tuberous sclerosis complex brain lesions confirming the cell-specific activation of the mTOR pathway in cortical tubers (Boer et al., 2008). Other studies have investigated a role for mTOR in oligodendrocyte differentiation and CNS myelination and have demonstrated that mTOR is vital for these processes. Mice with oligodendrocyte-specific knockdown of mTOR in the CNS using Cre-recombinase under control of the 2–3-cyclic nucleotide 3-phosphodiesterase (CNP) promoter show reduced oligodendrocytes and myelination impairments in the spinal cord (Wahl et al., 2014).

Another interesting possibility is that cytokine-driven T cell proliferation likely contributes to the induction of MS, as well as motor impairment in EAE models (Huseby et al., 2012). Recent evidence has suggested that inhibition of PI3K signaling could weaken the immune response via suppression of pro-inflammatory cytokine secretion, pointing to a potential therapeutic target in treating inflammatory diseases (Xie et al., 2014). Compelling data also suggests that the mTOR inhibitor, rapamycin, clinically used as an immunosuppressant, selectively inhibits activation of T-helper cell subsets, ultimately suppressing the mTOR-signal transducer and activator of transcription 3 (STAT3) pathway (Hou et al., 2017). This reduces protein and mRNA levels of interferon and interleukin in the spinal cord of EAE mice, thus promoting immunosuppression (Hou et al., 2017).

MS was believed to be a painless disease, however 50%–80% of MS patients report the presence of pain in their life (Nick et al., 2012, Thibault et al., 2011, Truini et al., 2011), which can broadly be classified as nociceptive or neuropathic (O'Connor et al., 2008). Given that glial cells contribute to the development and maintenance of pain hypersensitivity following nerve lesion (Ferrini and De Koninck, 2013), enhanced mTOR signaling may contribute to the persistent pain of MS through regulation of specific cellular activities. Repeated administration of rapamycin following the induction of EAE, significantly reduced mechanical allodynia and offered protection from developing severe motor impairment (Lisi et al., 2012). However, other studies have found that the PI3K/Akt/mTOR pathway is downregulated following the onset of EAE, which is restored with the administration of cannabinoids (Giacoppo et al., 2017). This occurs through cannabinoid type 1 (CB1) receptor activation of the pathway, promoting cellular survival in astrocytes and oligodendrocytes while inhibiting production of cytokines.

Preclinical studies have identified that EAE rodents develop mechanical hyperalgesia and cold allodynia following onset of the disease (Solaro et al., 2013, Thibault et al., 2011). In MS patients, it seems that neuropathic pain may be the direct result of central demyelination (Thibault et al., 2011). However, the exact molecular underlay of this comorbidity is not well understood. Using an EAE-recovered mouse model, one group found that pain reactivated inflammation through the accumulation of immune cells, such as the pathogenic CD4^+^ T cells, at specific sites on the spinal cord (Arima et al., 2015). The inhibition of mTOR kinase activity in neuroimmune cells results in anti-inflammatory actions, suggesting possible beneficial effects of mTOR inhibitors (like rapamycin), however mTOR plays an important role in the regulation of oligodendrocyte development and myelination. This would limit its therapeutic potential, especially for conditions such as MS. Additional studies are needed to determine the true role of mTOR in MS-related neuropathic pain and whether benefits may be observed through a direct mTOR activation mechanism within spinal pain (or brain) transmission pathways (Asante et al., 2009, Asante et al., 2010, Geranton et al., 2009).

## Depression

Major depressive disorder (MDD) is the most common mental illness, affecting about 5% of the population every year (Abelaira et al., 2014). It is often characterized by decreased mood, changes in previously enjoyable activities, alterations in appetite and sleep and suicidal ideation (Abelaira et al., 2014). Despite recent advances in understanding monoamine dysfunction in MDD, the biological mechanisms underlying this mental health disorder remain poorly understood. Typical treatment regimens for MDD require lengthy treatment courses to establish antidepressant effects, which suggest that aberrant neural plasticity is a mitigating factor. This has prompted a deeper exploration of the molecular mechanisms associated with depression, focusing on regulators of neural plasticity. Specifically, using a rodent model of depression, Li et al. (2010) demonstrated that administration of the dissociative anesthetic ketamine, a glutamate N-methyl-D-aspartic acid receptor (NMDAR) antagonist, rapidly activated the mTOR pathway, leading to increased synaptic signaling and new spine synapses in the prefrontal cortex (PFC). Moreover, a single administration of ketamine was sufficient to reverse the depressive phenotype and induction of synaptogenesis observed in the rodent models, with similar reversal seen in human clinical trials (Aleksandrova et al., 2017, Li et al., 2010). Further, mTOR phosphorylation and activation were decreased in the brains – PFC, amygdala, and hippocampus – of postmortem MDD patients, supporting a role for dysfunctional mTOR signaling in the pathology of MDD (Ignacio et al., 2016).

In approximately 30%–50% of chronic pain patients, MDD is a common comorbidity (Barthas et al., 2017, Xie et al., 2017). The molecular mechanisms underlying this relationship remain unclear, but point toward the influence of inflammatory and neurotrophic factors, although the latter is less well-understood (Sheng et al., 2017, Xie et al., 2017). In response to nerve injury and inflammation, brain derived neurotrophic factor (BDNF) is released from microglia in the spinal cord (Beggs et al., 2012, Coull et al., 2005) and possibly the brain (Taylor et al., 2015) resulting in neuronal hyperexcitability. These changes disrupt important neurotransmitter pathways resulting in increased NMDAR excitation, excessive levels of glutamate and switching inhibitory neurotransmission to excitatory (Walker et al., 2014). This is thought to affect mood as supported by transient changes in cognition and mood in healthy subjects given an inflammatory stimulus (Walker et al., 2014). Evidence demonstrates that BDNF is altered in chronic pain animals in areas specific to MDD/emotion processing, including the PFC, hippocampus and amygdala (Ishikawa et al., 2015, Xie et al., 2017, Yu and Chen, 2011). However, this depressive phenotype and pain hypersensitivity can be reversed with administration of ketamine, which is known to normalize pro-inflammatory cytokine levels (Xie et al., 2017).

While ketamine is known to be an antagonist of the NMDAR and glutamatergic transmission, it also affects HCN1 channels, cholinergic, aminergic and opioid systems (Zorumski et al., 2016). Ketamine’s antidepressant effects far outlast detectable drug levels suggesting that they may be mediated by a secondary increase in structural synaptic connectivity and plasticity. In addition, ketamine causes a disinhibition of glutamatergic transmission through an α-amino-3-hydroxy-5-methyl-4-isoxazolepropionic acid receptor (AMPAR)-dependent mechanism, resulting in synaptogenic effects (Ignacio et al., 2016). Specifically, preclinical studies demonstrated that rats treated with ketamine displayed decreased FST immobility in tandem with increased hippocampal and PFC BDNF expression and increased phosphorylated mTOR levels (Yang et al., 2013, Zhou et al., 2014). Further, administration of rapamycin in tandem with ketamine, blocked the antidepressant effects of ketamine and actually induced cognitive deficits (Holubova et al., 2016). On its own, sub-chronic administration of rapamycin at high doses alleviated affective symptoms (Cleary et al., 2008, Russo et al., 2013), but had no effect or induced depressive symptoms with chronic administration (Russo et al., 2013). Interestingly, delivering an AMPAR agonist before ketamine enhanced the antidepressant effects of ketamine compared to an AMPAR antagonist, supporting an integral role for AMPAR in ketamine’s antidepressant effects (Zhou et al., 2014). Together these data suggest that AMPAR activation, mTOR, and BDNF may create a positive feedback loop in discrete brain regions, which contributes to various mood disorders and possibly persistent pain signaling (Ignacio et al., 2016, Yang et al., 2013, Zhou et al., 2014).

## Alzheimer’s disease

Dementia represents the most prevalent form of neurodegeneration (Alzheimer's, 2016, Hirtz et al., 2007, Wong et al., 2016) with Alzheimer’s disease (AD) accounting for approximately 60% of all dementia cases (Lobo et al., 2000). The pathological hallmarks of AD include amyloid plaques derived from Aβ accumulation, neurofibrillary tangles derived from tau hyperphosphorylation and synapse loss, which are altogether associated with concurrent memory impairment (Masters et al., 2015). These cognitive dysfunctions are correlated with notable degeneration in the transentorhinal cortex and hippocampus (Nelson et al., 2009).

The mTOR signaling pathway has been heavily implicated in AD. It was found to be involved in both Aβ and tau pathologies through increased activity in mostly memory-related brain regions (Galvan and Hart, 2016). A number of reports indicate that mTOR phosphorylation at Ser^2481^ and Ser^2488^ was increased in homogenate of medial temporal cortex and the inferior parietal lobule of AD cases (Griffin et al., 2005, Li et al., 2005, Tramutola et al., 2015). Increased mTOR coincided with neurons affected by tau pathology (An et al., 2003, Iyer et al., 2014, Ma et al., 2010). Also, enhanced phosphorylation of PRAS40, a subunit of mTORC1, was found in the triple-transgenic mouse model of AD (3xTG-AD) that exhibit both Aβ and tau pathologies (Caccamo et al., 2011). An increase in mTOR activity can partly be explained by elevated levels of zinc, an upstream activator of mTOR, in the hippocampus and amygdala (An et al., 2003, Cornett et al., 1998, Danscher et al., 1997, Deibel et al., 1996). Also, Aβ accumulation was shown *in vitro* and *in vivo* to trigger mTOR hyperactivity through the PI3K/Akt pathway (Oddo, 2012). In line with this, in 3xTG-AD mice, mTOR hyperactivity could be stopped and normalized upon administration of an anti-Aβ antibody (Caccamo et al., 2011). Furthermore, in 3xTG-AD mice, mTOR activity was increased in the cortex and hippocampus in an age-dependent manner (Caccamo et al., 2010, Caccamo et al., 2011). In humans, phosphorylation of PI3K p85α subunit at the Tyr^508^ residue and Akt at the Ser^473^ residue were detected during the early stages of mild cognitive impairments, indicating involvement of mTOR in the development of AD. Increased mTOR was found to lead to increased phosphorylation of its downstream targets including p70S6K and eIF4E (An et al., 2003, Chang et al., 2002, Griffin et al., 2005, Onuki et al., 2004, Peel and Bredesen, 2003, Pei et al., 2008, Pei and Hugon, 2008). In response to elevated levels of mTOR, administration of rapamycin was shown to decrease the pathological hallmarks of AD and associated cognitive deficits in 3xTg-AD, Tg2576 and hAPP (J20) mouse models (Caccamo et al., 2014, Caccamo et al., 2010, Harrison et al., 2009, Lin et al., 2013, Spilman et al., 2010, Wilkinson et al., 2012). Such improvements are brought on in part by downregulation of tau translation and hyperphosphorylation at multiple serine/threonine residues as was found in the perforant pathway and by the activation of autophagy in brain parenchyma (Caccamo et al., 2010, Galvan and Hart, 2016, Siman et al., 2015, Spilman et al., 2010, Tang et al., 2015, Tepper et al., 2014).

Interest has grown regarding the assessment and management of pain in AD (Gagliese, 2009) and it has been estimated that 50–93% of AD patients suffer from a pain condition (Abdulla et al., 2013, Corbett et al., 2014, van Kooten et al., 2015, Whitlock et al., 2017). Neurodegenerative changes – often observed in AD – have been shown to occur in brain regions associated with pain processing including the locus coeruleus, hypothalamus, periaqueductal grey, anterior cingulate cortex, insula and amygdala (Cole et al., 2006, Scherder et al., 2003, Stubbs et al., 2016, Zarow et al., 2003). Progressive decline in cognitive function makes self-assessment of pain unreliable and has led to contradictory observations (Defrin et al., 2015, Scherder et al., 2003, Wynne et al., 2000). On the other hand, experimental studies based on observations of pain responses (i.e. facial, motor and brain) have found increased responsiveness in individuals with early to moderate AD symptoms compared with healthy controls (Cole et al., 2006, Cole et al., 2011, Hadjistavropoulos et al., 2000, Kunz et al., 2007, Lints-Martindale et al., 2007). Individuals with AD displayed an increased facial response, lowered threshold for the nociceptive flexion reflex, and elevated/prolonged brain activities in pain pathways and other regions including dorsolateral prefrontal cortex (Defrin et al., 2015). However, it remains to be determined whether similar changes occur in advanced AD cases. We suspect that altered pain processing occurs, in part, as a result of mTOR dysfunction, which is not limited to the temporal regions of the brain, but spreads throughout the pain processing regions of the brain as the neurodegeneration progresses.

## Parkinson’s disease

Parkinson’s disease (PD) represents the second most common neurodegenerative disorder characterized by classical parkinsonian motor symptoms that include muscular rigidity, bradykinesia, rest tremor, and postural instability (Kalia and Lang, 2015). PD pathology is marked by progressive loss of dopaminergic neurons in the substantia nigra pars compacta (SNpc), a component of the basal ganglia complex that includes the striatum (Blandini et al., 2000, Kalia and Lang, 2015).

Several studies have observed dysregulation of mTOR in PD albeit the directionality of change remains controversial (Lan et al., 2017). Elevated levels of mTOR have been found in the striatum and SNpc of PD brains (Dijkstra et al., 2015, Wills et al., 2012). Similar upregulation of mTOR was found in ventral midbrain and dopaminergic neurons of SNpc in engrailed 1 null mutant mice (*En1*^+/−^) where En1 is a transcription factor implicated in the survival of mesencephalic dopaminergic neurons (Nordstroma et al., 2015). Also, administration of environmental risk factors for PD, maneb and paraquat, were shown to increase mTOR levels in the striatum of wild-type mice (Wills et al., 2012).

In contrast to these findings, several studies have proposed that mTOR plays a protective role in PD. For instance, mTOR activity was found to decrease upon administration of neurotoxins that causes permanent symptoms of PD such as 6-hydroxydopamine (6-OHDA), 1-methyl-4-phenyl-1,2,36-tetrahydropyridine (MPTP) and rotenone (Chen et al., 2010, Rieker et al., 2011, Rodriguez-Blanco et al., 2012, Selvaraj et al., 2012, Xu et al., 2014, Zhou et al., 2015). Also, upon transduction of constitutively active and myristoylated Akt, in the dopaminergic neurons of the SNpc leads to axonal regeneration following a neurotoxin-mediated lesion (Kim et al., 2011). In line with this, an upstream activator of mTOR, Tnfaip8/Oxi-α, was found to protect dopamine neurons from oxidative stress-induced cell death by activating mTOR kinase and the subsequent phosphorylation of p70 S6 kinase and 4E-BP1/2 (Choi et al., 2010). Furthermore, in humans, an upstream inhibitor of mTORC1 called REDD1 is highly expressed in SNpc of PD patients and is reduced upon rapamycin administration (Malagelada et al., 2006).

In support of the human literature showing elevated levels of mTOR, administration of rapamycin activates the translation inhibitor, 4E-BP1/2, which leads to prevention of apoptosis, enhancement of autophagy and protection against α-synuclein accumulation in *in vitro* and *in vivo* models of PD (Bockaert and Marin, 2015). Furthermore, in animal models of PD, inhibition of mTOR using rapamycin or Temsirolimus (CCI-779) were able to prevent dyskinesia triggered by long-term usage of l-DOPA, a treatment used to manage motor symptoms in PD (Decressac and Bjorklund, 2013, Santini et al., 2009, Subramaniam et al., 2011). l-DOPA is known to activate mTOR through Rhes, a striatum-specific Rheb ortholog (Subramaniam et al., 2011). There are also indications that additional compounds including a statin-derivative (rosuvastatin), mesencephalic astrocyte-derived neurotrophic factor and ketamine provided neuroprotective effect on dopaminergic neurons of SNpc via a reduction in mTOR (Fan et al., 2017, Kang et al., 2017, Zhang et al., 2017).

It is estimated that 40–95% of individuals with PD also suffer from persistent nociceptive or neuropathic pain that can be derived from PD itself or from secondary disorders that are of musculoskeletal or visceral origin (Avenali et al., 2017, Broen et al., 2012, Buhmann et al., 2017, Chaudhuri et al., 2006). Specifically, the majority of PD patients suffer from back or joint pain (Avenali et al., 2017). Also, pain hypersensitivity and lower pain thresholds in response to electrical and heat stimuli are reported in PD (Mylius et al., 2009). These alterations in pain sensitivity and the prevalence of pain conditions in PD can be explained by the neurodegenerative changes in the functional connectivity between basal ganglia and pain processing regions such as thalamus, frontal and parietal lobes, insula and hippocampus (Chudler and Dong, 1995, Pertovaara and Wei, 2008, Potvin et al., 2009, Skogar and Lokk, 2016). As dopaminergic projections from the SNpc and ventral tegmental area innervate brain regions that overlap with pain processing, the neurodegeneration observed in PD will likely lead to changes in motor and sensory perceptions (Braz et al., 2005, Porro et al., 1999, Simon et al., 1979). Basal ganglia neurons are implicated in nociception through their descending adrenal-dopaminergic and GABAergic inhibitory projections (Avenali et al., 2017, Pertovaara and Wei, 2008, Potvin et al., 2009, Tassorelli et al., 2007). As part of the basal ganglia, SNpc neurons are also known to respond to nociceptive stimuli under normal conditions in rodents and primates (Romo and Schultz, 1989, Tassorelli et al., 2007), and its activation can lead to hyposensitivity to pain as demonstrated using hot plate and tail flick tests (Jurna et al., 1978, Sandberg and Segal, 1978, Tassorelli et al., 2007).

## Implications for brain mechanisms of chronic pain

Chronic pain involves many, still poorly understood, pathophysiological processes in the nervous system that produce and exacerbate suffering. These processes involve abnormal neural plasticity in the spinal cord and brain, switching of neuronal phenotypes, and uninjured nerves acquiring the function of injured ones. In comparison with the DRG and spinal cord, the molecular changes that occur in higher brain structures that are associated with the maintenance of pain symptoms are less clear. In fact, most of the evidence for supraspinal neurodegeneration in chronic pain comes from human brain imaging studies. This review has touched upon some – but by no means all – of the neurological diseases that exhibit changes in mTOR-related brain activity (Table 1). These changes were limited to the brain regions known to play a critical role in each respective disorder, but since pain is a common comorbidity for these diseases, we suspect that the induction of mTOR activity by neurodegenerative diseases in pain associated areas might lead to aberrant plasticity that causes pain symptoms.

**Table 1:** Summary of mTOR and pain implications in various neurological disorders.

Disorder	Key features	mTOR implications	Pain implications	Brain regions
Multiple Sclerosis (MS)	• Autoimmune disorder (chronic inflammation) • Scattered areas of lesions or plaques in the CNS • Demyelination, microglia activation, gliosis, axonal damage	• mTOR knockdown leads to reduced oligodendrocytes and myelination impairments of the spinal cord • mTOR phosphorylation leads to pro-inflammatory microglia activation • Suppressing mTOR-STAT3 pathway could promote immunosuppression	• 50%-80% of MS patients experience pain • Enhanced mTOR signaling could promote pain in MS patients • Pain reactivates spinal inflammation via CD4^+^ T cells • Rapamycin and targeting cannabinoid receptors may be used as a pain therapy	• Not explicitly known

Major Depression Disorder (MDD)	• Mental illness • Decreased mood and changes in pleasurable activities	• mTOR phosphorylation and activation is decreased in MDD patients • Ketamine administration can lead to rapid activation of the mTOR pathway causing increased synaptic signaling and new spine synapses leading to decreased depressive phenotype	• 30%-50% of MDD patients experience pain as a comorbidity • BDNF is released from microglia in response to injury and/or inflammation causing neuronal hypersensitivity and excitability leading to enhanced pain sensations • BDNF is altered in chronic pain patients in the brain regions implicated in MDD. • Ketamine administration reverses pain hypersensitivity and depressive phenotype and increases hippocampal and PFC BDNF • Potential feedback loop of AMPAR, mTOR, and BDNF in contribution to MDD and persistent pain	• PFC (Aleksandrova et al., 2017, Ignacio et al., 2016, Ishikawa et al., 2015, Li et al., 2010, Yu and Chen, 2011, Xie et al., 2017) • Amygdala (Ignacio et al., 2016, Ishikawa et al., 2015, Yu and Chen, 2011, Xie et al., 2017) • Hippocampus (Ignacio et al., 2016, Ishikawa et al., 2015, Yu and Chen, 2011, Xie et al., 2017)

Alzheimer’s Disease (AD)	• Represents 60% of all dementia cases • Pathological hallmarks are amyloid plaques and neurofibrillary tangles • Progressive memory impairment	• Increases in mTOR coincides with both Aβ and tau pathologies • Increases in mTOR signaling observed in both humans and animal models • Rapamycin reduces AD pathologies and associated cognitive deficits	• 50–93% of patients suffer from a pain condition • Neurodegeneration occurs in brain regions implicated in pain processing • Increased responsiveness to pain was observed in early to moderate AD patients	• Temporal cortex (Griffin et al. (2005), Li et al. (2005)) • Forebrain (Caccamo et al., 2010, Caccamo et al., 2011, Caccamo et al., 2014))

Parkinson’s Disease (PD)	• Second most common neurodegenerative disorder • Progressive loss of dopaminergic neurons in SNpc • Motor symptoms include muscular rigidity, bradykinesia, rest tremor and postural instability	• Increases in mTOR signaling found in both humans and animal models • In contrast, several studies using animal models and cell lines posit mTOR plays a protective role • Rapamycin protects against α-synuclein accumulation and prevent L-DOPA-triggered dyskinesia	• 40–95% of patients suffer from persistent nociceptive or neuropathic pain • Majority suffers from back or joint pain • Pain hypersensitivity and lower pain thresholds against electrical and heat stimuli were reported	• Striatum (Dijkstra et al. (2015), Wills et al. (2012)) • SNpc, VTA (Rieker et al. (2011), Selvaraj et al. (2012))

The involvement of mTOR in augmenting neural plasticity in the brain following chronic pain is not known, but at least one study has shown that hind paw injections of bee venom increased mTOR phosphorylation and enhanced LTP in the entorhinal cortex-CA1 pathway, both of which were reversed by pre-treatment with rapamycin (Lyu et al., 2013). While the behavioral effects of these changes remain uncertain, they suggest that altered mTOR signaling in discrete brain regions may drive the cognitive and/or emotional aspects of pain. It has recently been shown that following nerve injury the mTOR pathway is enhanced in the insular cortex (IC) and direct infusion of rapamycin into the IC reversed mechanical allodynia in rats (Kwon et al., 2017). The IC is involved in sensory and cognitive processes and exhibits connectivity with other regions to influence higher-level functions, such as pain-perception and decision-making (Zhuo, 2016). In fact, the small study by Kwon et al. (2017) may only represent the tip of the iceberg and pain may alter mTOR signaling to change the affective, motivational and sensory functions of the brain.

## Concluding remarks

The dysregulation of mTOR in a wide array of neurological disorders highlights the complexity of this signaling pathway (summarized in Table 1) and suggests that normal mTOR activity is essential for optimal health. Given the essential role of mTOR in cell survival and protein translation, it should probably not be considered an ideal drug candidate. Inhibition of mTOR signaling using rapalogs, including rapamycin produces debilitating and serious side-effects such as rash, anemia, fatigue, hyperglycemia, decreased appetite, nausea, and diarrhea (Pallet and Legendre, 2013). As physiological levels of mTOR are important for neuronal health, a homeostatic balance with regards to mTOR activity must be maintained rather than complete inhibition or overexpression. However, continuous efforts should be made to identify therapeutic downstream targets of this key signaling pathway that are specific to the given disorder and/or pain condition. We currently do not know whether modulating the mTOR pathway in the brain is sufficient to reverse chronic pain-related symptoms including hypersensitivity or comorbidities including anxiety and depression. A study is needed to evaluate the specific changes of mTOR activity in the brain as a consequence of chronic pain, but different types of pain – cancer, inflammatory and neuropathic – should be evaluated because the role of mTOR is likely to be different depending on the underlying cause(s) of pain. A greater understanding of these signaling pathways will give great insight into disease mechanism and possibly lead to a more refined therapeutic approach.

## Conflict of interest

The authors declare that they have no conflicts of interest.
